# Impact of Emotional Intelligence on Stress Levels Among Medical Students

**DOI:** 10.1192/j.eurpsy.2025.1574

**Published:** 2025-08-26

**Authors:** K. Imen, R. Nakhli, L. S. Chaibi, C. Sridi, M. Bouhoula, N. Belhadj, F. Chelly, A. Aloui, M. Kahloul

**Affiliations:** 1 Occupational Medicine Department, University of Sousse, Faculty of Medicine Ibn Al Jazzar; 2 2. Occupational Medicine Department, 1. University of Sousse, Faculty of Medicine Ibn Al Jazzar, Sousse; 3 D Psychiatric Department, Razi Hospital, Tunis; 4 Occupational Medicine Department, Occupational Medicine Department, Sahloul University Hospital; 5 4. Anesthesia and Intensive Care Department, Sahloul University Hospital, Sousse, Tunisia

## Abstract

**Introduction:**

The prevalence of stress and emotional disorders among medical students has risen dramatically in recent years. In response, it is essential to develop prevention strategies, such as emotional intelligence (EI), to better manage stress and preserve mental well-being.

**Objectives:**

To evaluate the level of EI among medical students and study its relationship with perceived stress.

**Methods:**

This cross-sectional, analytical study was conducted among medical students at the Faculty of Medicine, Sousse, from March 2023 to February 2024. Participants completed a questionnaire assessing perceived stress using the Perceived Stress Scale (PSS10), emotional intelligence using the Schutte Emotional Intelligence Test, and lifestyle habits including sports participation and psychoactive substance use.

**Results:**

The study included 207 students, with a majority being female (87.7%). Among them, 88.4% had no psychiatric history. Alcohol consumption was noted in 36 students, while 10 participants reported cannabis use. Regular physical activity was observed in 64.3% of cases. Students with lower EI scores generally exhibited higher stress levels, especially in the absence of sports or extracurricular activities. While correlations between EI and stress were evident, univariate analysis did not reveal a statistically significant direct association (p=0.416). Psychiatric symptoms, including anxiety and emotion regulation difficulties, emerged as significant factors in stress modulation, independent of EI levels.emotion regulation appeared to play a major role in stress modulation, independent of EI.

**Conclusions:**

EI emerges as a critical factor in enhancing resilience against stress among medical students. By developing a more profound understanding and effective regulation of emotions, students can significantly elevate their mental and emotional well-being.

**Disclosure of Interest:**

None Declared

